# Assessing and Enhancing Nutrition and Physical Activity Environments in Early Childhood Education and Care Centers: Scoping Review of eHealth Tools

**DOI:** 10.2196/68372

**Published:** 2025-01-22

**Authors:** Joyce Hayek, Kelsi Dickson, Lynne M Z Lafave

**Affiliations:** 1 Department of Health and Physical Education Mount Royal University Calgary, AB Canada

**Keywords:** eHealth, early childhood educators, ECE, early childhood education and care, ECEC, knowledge synthesis, digital technology, health technology, digital public health, eating, diet

## Abstract

**Background:**

Early childhood is a critical period for shaping lifelong health behaviors, making early childhood education and care (ECEC) environments ideal for implementing nutrition and physical activity interventions. eHealth tools are increasingly utilized in ECEC settings due to their accessibility, scalability, and cost-effectiveness, demonstrating promise in enhancing educators’ practices. Despite the potential effectiveness of these eHealth approaches, a comprehensive collection of available evidence on eHealth tools designed to assess or support best practices for nutrition or physical activity in ECECs is currently lacking.

**Objective:**

The primary objective of this scoping review is to map the range of available eHealth tools designed to assess or deliver interventions aimed at improving nutrition or physical activity in ECEC settings, while evaluating their components, theoretical foundations, and effectiveness.

**Methods:**

This scoping review adhered to the Joanna Briggs Institute methodology, in accordance with the PRISMA-ScR (Preferred Reporting Items for Systematic Reviews and Meta-Analyses Extension for Scoping Reviews) checklist. The objectives, inclusion criteria, and methods for this review were predefined and specified. Eligibility criteria were (1) early childhood educators (population); (2) eHealth (digital) technologies, such as websites, smartphone apps, emails, and social media; and (3) tools designed to assess or deliver interventions aimed at improving best practices for nutrition, physical activity, or both within ECEC settings (context). A search was conducted across 5 electronic databases (PubMed, Scopus, CINAHL Plus, ERIC, and Embase) to identify white literature, and 3 electronic databases (ProQuest, Google Scholar, and targeted Google search), along with hand-searching of reference lists, were used to identify gray literature. All literature was reported in English or French, with the search extending until May 2024. Separate data charting tools were used for white and gray literature.

**Results:**

The search strategy identified 3064 results for white literature, yielding 2653 unique citations after duplicates were removed. Full texts for 65 citations were retrieved and screened for inclusion, resulting in 30 studies eligible for data extraction and analysis. The most common study design was a randomized controlled trial, comprising 16 studies (53%). The largest proportion of studies were conducted in the United States (11 studies, 37%). In total, 19 eHealth tools were identified, targeting nutrition (8 tools, 42%), physical activity (5 tools, 26%), or both nutrition and physical activity (6 tools, 32%). All tools were web based (19 tools, 100%). The gray literature search yielded 1054 results, of which 17 were moved to full-text screening, and 7 met the eligibility criteria for data extraction and analysis. The tools identified in the gray literature originated in Canada (4 tools, 57%) and the United States (3 tools, 43%). The majority targeted nutrition (4 tools, 57%) and were primarily web based (6 tools, 86%), with 1 mobile app (1 tool, 14%).

**Conclusions:**

This scoping review mapped the available eHealth tools designed to improve nutrition or physical activity environments in ECEC settings, highlighting the growing emphasis on web-based tools and the need for psychometric testing. Future research should systematically evaluate the effectiveness of these tools, particularly those addressing both nutrition and physical activity, to identify the key factors that contribute to long-term behavior change.

**Trial Registration:**

Open Science Framework XTRNZ; https://osf.io/xtrnz

**International Registered Report Identifier (IRRID):**

RR2-10.2196/52252

## Introduction

### Background

Unhealthy lifestyle factors, such as physical inactivity and unhealthy diets, are primary contributors to the rising incidence of chronic diseases [1-5]. These conditions are recognized as growing global public health problems, leading to significant treatment costs and imposing an economic burden on health systems, individuals, and society as a whole [6,7]. Health behaviors often originate in early childhood and can persist into young adulthood [8]. Research indicates that health behaviors, such as eating habits and physical activity levels, are modifiable risk factors for obesity and chronic diseases [9]. These behaviors often co-occur or cluster together [10,11]. An integrated approach to health promotion, addressing both dietary intake and physical activity simultaneously [10], is therefore essential during early childhood to improve population health across the lifespan.

The early years are a critical period for shaping health behaviors and outcomes [12], with early interventions regarded as an essential component of preventive health [13]. Early childhood education and care (ECEC) settings provide a unique opportunity to reach a large number of children during this pivotal developmental period, making them an ideal setting for behavioral interventions [12,14]. Moreover, early childhood educators, as part of the ECEC environment, are well-positioned to successfully implement nutrition and physical activity behavior interventions [15,16]. Evidence from prior research shows that professional training of early childhood educators in best practices for nutrition and physical activity is associated with improved dietary intake and increased physical activity levels in young children [17-20].

With the widespread use of the internet, online or eHealth interventions have experienced significant growth. eHealth refers to the use of digital technologies, such as the internet, digital gaming, virtual reality, and robotics, for promoting, preventing, treating, and maintaining health [21]. Examples of eHealth technologies include smartphone apps, websites, computer programs, SMS text messaging, and social media platforms [22]. Digital technologies offer several advantages, including lower costs, reduced participant burden, enhanced accessibility, and increased scalability, thereby extending the reach of behavioral interventions [23,24]. Prior research indicates that eHealth interventions within ECEC settings are highly acceptable and effective in improving early childhood educators’ knowledge and practices related to nutrition and physical activity [25-27].

Behavior change theories can guide the selection of intrapersonal constructs to target in intervention development, as well as the choice of behavior change techniques to achieve desired behavioral outcomes [28]. Evidence suggests that behavior change interventions, whether internet-based or not, are more effective when guided by a theoretical framework [29]. A meta-analysis [29] found that interventions extensively informed by theory demonstrated larger effects compared with those lacking theoretical underpinnings. The use of theory not only enhances the efficacy of interventions but also facilitates their replication and future development [30]. Consequently, it is crucial to determine whether theory has been applied in the development of eHealth tools.

Recognizing the importance of early learning settings and educators in fostering healthy behavior development in children, researchers have developed, implemented, and evaluated health promotion interventions within childcare settings [31-33]. A preliminary search of PROSPERO, PubMed, the Cochrane Database of Systematic Reviews, and JBI Evidence Synthesis identified several relevant reviews. However, these reviews primarily focused on evaluating the effectiveness of in-person nutrition and physical activity interventions [34,35], interventions conducted in family-based centers [36,37], or those targeting older children and adolescents [33]. No current or in-progress systematic or scoping reviews address eHealth tools for promoting the best nutrition and physical activity practices in ECEC settings.

### Objectives

To address this gap in the literature, we conducted a scoping review. This method is used to identify the types of available evidence in a field, explore key characteristics related to a concept, and analyze knowledge gaps [38]. This review aimed to achieve the following objectives: (1) identify existing eHealth tools used to assess or deliver interventions that improve nutrition or physical activity environments in ECEC centers; (2) describe the components of the eHealth tools, including technology type (eg, websites, smartphone apps, social media) and health purposes (eg, nutrition evaluation, physical activity promotion); (3) outline the psychometric properties of the eHealth tools, when applicable; (4) report the theoretical foundations used in developing the eHealth tools; and (5) identify any evidence gaps. The purpose of this study was to map the available evidence on eHealth tools currently used to assess and support best practices for nutrition or physical activity in ECEC centers.

## Methods

### Overview

This scoping review followed established methods for such studies [39] and adhered to the PRISMA-ScR (Preferred Reporting Items for Systematic Reviews and Meta-Analyses Extension for Scoping Reviews) guidelines [40]. Methodological quality or risk of bias was not assessed, as the goal of this review was to provide a broad overview of existing evidence, regardless of methodological approach, to map the available evidence. This is consistent with guidance on scoping review methods [39]. Full details of the methods can be found in our published protocol [41]. The PRISMA-ScR checklist is included in Multimedia Appendix 1 [40].

### Selection Criteria

#### Participants

This review considered studies involving early childhood educators within licensed ECEC programs, whether public or privately operated, providing full-day care for children aged 0-5 years. Studies focusing on educators in family-based settings, preschool programs where children attend for less than 4 hours per day, or before- and after-school care were excluded.

#### Concept

This review considered studies that explored eHealth tools designed to support nutrition or physical activity environments in the ECEC setting. eHealth tools were defined as digital technologies that (1) assessed or (2) delivered interventions to improve nutrition or physical activity environments and practices. eHealth tools that assessed the ECEC environment were included only if they provided feedback to the ECECs. Additionally, we included only studies where the eHealth tool was the primary component for assessment or intervention.

#### Context

This review focused on nutrition and physical activity environments in the ECEC setting, considering both the physical and social environments.

#### Types of Sources

For this scoping review, we considered all study designs, including quantitative, qualitative, mixed methods, protocols, experimental, quasi-experimental, and cross-sectional studies. Systematic reviews and meta-analyses were also included if relevant to the topic, with a primary focus on ECEC settings. Unpublished studies and gray literature were also considered as sources of information.

### Search Strategy

#### Overview

The search strategy was developed in collaboration with 2 research librarians and aimed to capture both white and gray literature to encompass the full range of available eHealth tools.

#### White Literature

A preliminary limited search of PubMed and Scopus was conducted to identify articles on the topic. The text words in the title and abstract, along with the index terms used to describe the articles, were analyzed to develop a full search strategy for PubMed. This search strategy, incorporating all identified keywords and index terms, was then adapted for each included information source (see Multimedia Appendix 2). The databases searched included PubMed, Scopus, CINAHL Plus (EBSCOhost), ERIC (EBSCOhost), and Embase (OVID). The reference lists of all included sources of evidence were screened for additional white literature. The final search was conducted on October 4, 2023. For each database, a search alert was set up using its alert functions to track any new relevant publications for potential inclusion in the review. The final search alert was reviewed on May 5, 2024, and only 1 additional study was identified. All retrieved white literature was exported into Covidence software (Veritas Health Innovation) for screening and data extraction.

#### Gray Literature

Following the guidelines outlined by Godin et al [100], we conducted a thorough search of the gray literature to identify nonindexed sources such as dissertation abstracts, government documents, conference proceedings, educational materials, and reports. This was done through searches in (1) the ProQuest Database, (2) Google Scholar, (3) targeted web-based Google searches, and (4) hand searches of the reference lists of all included gray literature to identify additional relevant sources. The final gray literature search was conducted on April 30, 2024. All retrieved documents were exported into Microsoft Excel and assigned a unique identifier for screening and data extraction. Because of the potential volume of gray literature, we limited our review to the first 10 pages from Google Scholar and targeted web-based Google searches, based on title. Additionally, eHealth tools that required payment for access were excluded.

This review included studies and records published in English or French, with no date limitations.

#### Study Selection

**Figure 1:**
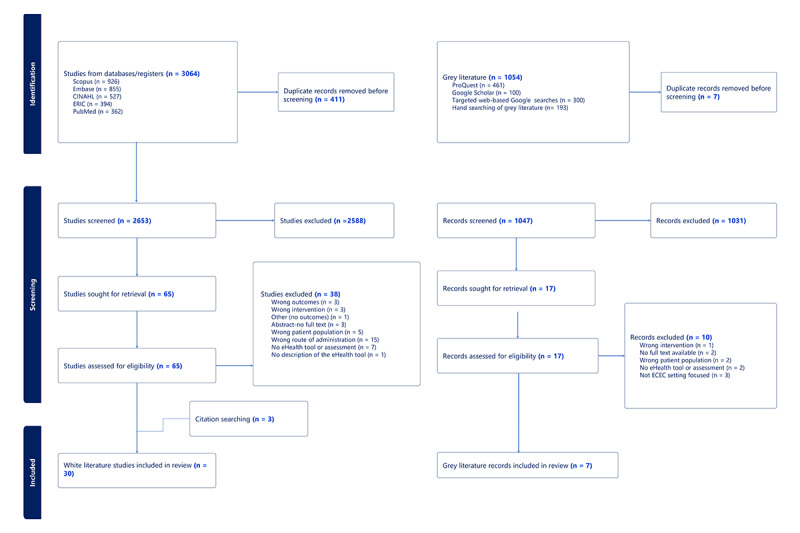
PRISMA (Preferred Reporting Items for Systematic Reviews and Meta-Analyses) flowchart.

Following the search, all identified records were uploaded into Covidence (for white literature) or Microsoft Excel (for gray literature). Title and abstract screening, full-text screening, and data extraction were performed by 2 independent reviewers (JH and LMZL) according to the inclusion criteria. Reasons for excluding full texts that did not meet the inclusion criteria were recorded and reported in the scoping review. Any disagreements between the 2 reviewers were resolved through discussion, and if consensus could not be reached, a third reviewer (KD) was consulted. The results of the search are reported and presented in a PRISMA-ScR flow diagram (Figure 1 in Results section). 

#### Data Extraction

Separate data extraction charting tools for white and gray literature were developed by the reviewers (Multimedia Appendix 3), as outlined in our published protocol [41]. The extraction tools, used to capture relevant study and eHealth tool characteristics, were piloted before the review to ensure consistency in information collection. Any disagreements between reviewers were resolved through discussion.

#### Data Analysis and Presentation

The extracted data were summarized using descriptive statistics (ie, frequency counts). The results were organized by each review question, highlighting study characteristics, eHealth tool characteristics, and the use of a theoretical framework. The findings are presented in a narrative summary, complemented by tables, charts, and illustrations.

## Results

### Literature Search and Selection Process

The white literature search yielded 3064 results, of which 411 were removed as duplicates. Titles and abstracts for 2653 records were screened for eligibility, and 2588 were excluded. Of the remaining records, 65 full-text articles were reviewed against the eligibility criteria. A total of 30 articles were included for data extraction (Figure 1). The gray literature search yielded 1054 results, of which 7 were removed as duplicates. Titles and abstracts of 1047 records were assessed against the eligibility criteria, and 17 were moved to full-text screening. Of these, 7 met the inclusion criteria and were included in the analysis. The PRISMA flowchart shows the selection process.

### Included Records

**Figure 2:**
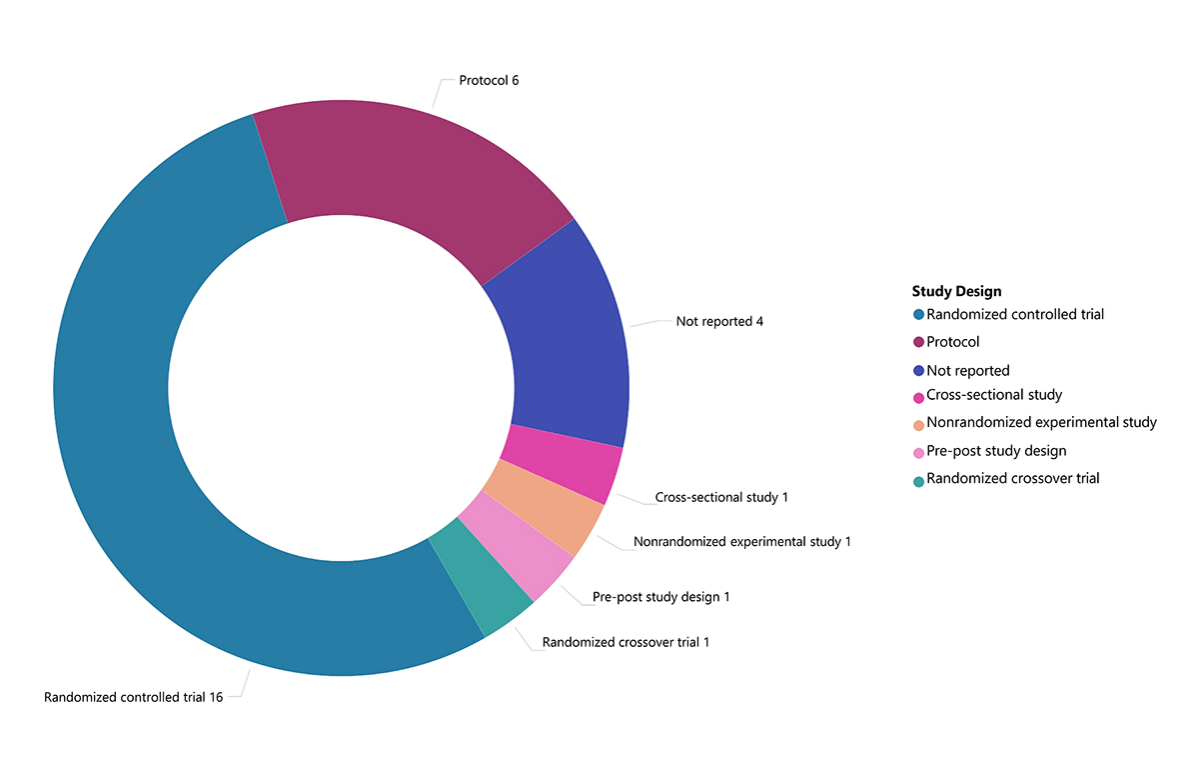
Study designs of research articles identified in the white literature.

**Figure 3:**
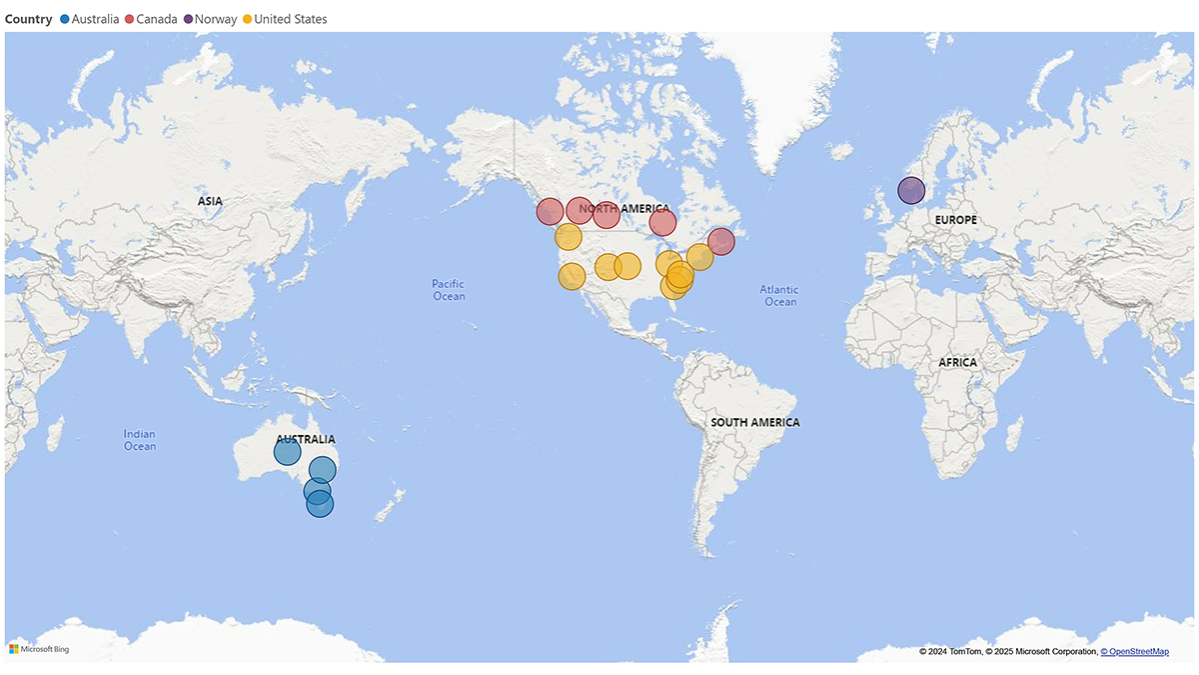
Country of study from eHealth research identified in the white literature and gray literature.

A total of 30 research studies identified in the white literature were included in this review (Figure 1). Of these, 16 were randomized controlled trials, 6 were protocol studies, 1 was a cross-sectional study, 1 was a nonrandomized experimental study, and 1 was a randomized crossover control trial (Figure 2). Specific characteristics of the included studies are presented in Table 1, which reflects the study design, setting, and outcome measures. The study aims along with additional study details are provided in Multimedia Appendix 4. The largest proportion of studies were conducted in the United States (n=11, 37%) and Australia (n=11, 37%), followed by Canada (n=6, 20%) and Norway (n=2, 7%; Figure 3). The studies were conducted between 2016 and 2022. The baseline sample size ranged from n=13 to 2932. Of the 10 studies reporting participant sex, females comprised the largest proportion of participants ranging from 85% to 100%, with an average of 96% (Table 1). The mean age of participants ranged from 37.1 to 49.6 years.

A total of 7 records were identified in the gray literature and included in this review (Figure 1). These records represented community-based eHealth outreach initiatives related to nutrition or physical activity support for the ECEC community. Four of these initiatives originated from Canada and 3 from the United States.

**Table 1:** Characteristics of included studies from the white literature.

Reference: country	Study design	Study setting	Sample size (at baseline)	Sex of overall participants (% of females)	Participant age (years), mean (SD)	Nutrition or PA^a^ outcome measures
Barnes et al [42]: Australia	RCT^b^	Childcare centers	NR^c^	NR	NR	•Improving implementation of targeted healthy eating practices. •Child dietary intake of fruit and vegetable servings in care. •Child dietary intake of sodium, saturated fat, and added sugar in care. •Mean servings of fruits and vegetables packed within lunch boxes.
Barnes et al [27]: Australia	RCT	Childcare centers	Total: 22 (intervention: 11; control: 11)	NR	Intervention group: supervisors 37.68 (5.92) and center champions 44.17 (6.40); control group: supervisors: 43.91 (10.57)	• Implementation of targeted healthy eating practices. • Supporting families to provide healthier foods consistent with dietary guidelines. •Provision of intentional healthy eating learning experiences. •Using feeding practices that support children’s healthy eating. •Staff participating in professional development targeting healthy eating. •Having a comprehensive written nutrition policy that outlines key healthy eating practices.
Blomkvist et al [43]: Norway	Protocol for RCT	Kindergarten	Total: 46 kindergartens (intervention: 31; control: 15)	NR	NR	• Primary outcomes: (1) child vegetable intake; (2) children’s level of food neophobia; and (3) child dietary habits and food variety. • Secondary outcomes: (1) self-reported weight and height; (2) parental and kindergarten staff feeding practices.
Blomkvist et al [44]: Norway	RCT	Kindergarten	Total: 46 kindergartens (intervention: 31; control: 15)	NR	NR	•Child intake of intervention vegetables and all vegetables combined. •Level of child food neophobia.
Bruijns^d,e^ et al [45]: Canada	Pre-post study design	Center-based childcare, kindergarten, and preschool	Total: 110 early childhood educators	99.2%	37.1 (9.5)	•Knowledge of PA, outdoor/risky play, and sedentary behavior concepts. •PA, outdoor/risky play, and sedentary behavior self-efficacy. •Behavioral intention and perceived behavioral control.
Brussoni et al [46]: Canada	Protocol for RCT	ELCC^f^	Total: 324 ECEs^g^ and ELCC administrators	NR	NR	•The primary outcome is increased tolerance of risk in children’s play, as measured by the Teacher Tolerance of Risk in Play Scale. •The secondary outcome is self-reported attainment of a self-developed behavior change goal.
Brussoni et al [47]: Canada	RCT	ELCC	Total: 563 educators and administrators (intervention: 281; control: 282)	96.6%	NR	•Primary outcome: change in the total score on the T-TRiPS^h^. •Secondary outcome: participants’ goal attainment at either follow-up time point (self-reported behavior change, measured by participants’ self-reported progress in attaining the goal they set for themselves).
Chuang et al [48]: United States	RCT	ECE centers	Total: 111 ECE providers (intervention: 56; control: 55)	97.3%	43.55 (11.87)	• Psychosocial and behavioral measures: (1) nutrition knowledge, (2) mindful eating, (3) perceived barriers to eating fruits and vegetables, and (4) perceived barriers to promote healthy eating in classroom. • Environmental factors: (1) nutrition-related policy and practices at their ECE facilities and (2) number of healthy eating–related activities organized for the staff in the ECE center. • Individuals behavior: (1) dietary intake and (2) communication of nutrition information with ECE children and parents.
Clark et al [49]: United States	NR	Licensed childcare	Total: 38 childcare providers (intervention: 23; control: 15)	100%	NR	• Knowledge of and attitudes and behaviors toward feeding breast milk, formula, and solid food to the infants in their care.
Clarke et al [50]: United States	Protocol	ECE programs	Total: 2932 ECE providers	N/A^i^	N/A	• Uptake and perceived usefulness of on-demand online nutrition training.
Grady et al [51]: Australia	Nonrandomized experimental study	Long day care services	Total: 46 childcare services (intervention: 27; control: 19)	NR	Intervention group: 48.44 (10.36); control group: 43.74 (10.48)	• Uptake and use of the menu program: proportion of services adopting the program and proportion of services using the program as intended.
Grady et al [52]: Australia	RCT	Long day care services	Total: 54 childcare services (intervention: 27; control: 27)	NR	Intervention group: 48.4 (10.4); control group 44.9 (10.5)	• Primary outcome: the mean number of food groups compliant with dietary guidelines •Secondary outcomes: (1) compliance with dietary guidelines for all food groups, (2) individual food group compliance with dietary guidelines, and (3) mean servings of individual food groups.
Green et al [53]: Australia	NR	Long day care, preschool, and occasional care	NR	NR	NR	• Nutrition practice achievements such as (1) lunch boxes monitored daily and (2) fruits and vegetables on menu. •PA practice achievements such as (1) tummy time for babies and (2) active playtime for at least 25% of opening hours.
Hazard et al [54]^e^: United States	RCT	Licensed childcare centers	Total: 20 childcare providers	98%	NR	• Evaluation of accessibility, acceptability, and satisfaction of nutrition and online education courses.
Hoffman et al [55]: United States	RCT	Preschools	Total: 11 teachers and 2 site supervisors	NR	NR	• Implementation fidelity, acceptability, and feasibility of WE PLAY^j^.
Hoffman et al [19]: United States	RCT	Preschools	Total: 25 teachers (intervention: 11; control: 14)	100%	Intervention group 40.7 (9.0); control group 45.9 (13.2)	• Children’s MVPA^k^ (MVPA accelerometer). •Teachers’ changes in PA knowledge and attitudes toward PA promotion.
Kempler et al [56]: Australia	Cross-sectional study	Childcare services	Total: 64 participants	NR	NR	• Qualitative outcomes explored use and experience with the menu tool.
Kennedy et al [57]: United States	NR	Preschools	Total: 41 teachers	NR	NR	•The percentage of the 60-minute daily goal reached in each classroom. •The proportion of students actively participating in MVPA. •The percentage of time spent in MVPA. •Teachers’ involvement with children during PA opportunities. •Child enjoyment (students having fun during the PA opportunities).
Lafave [58]: Canada	Randomized crossover trial design	ECEC^l^ centers	Total: 72 educators	100%	NR	• Psychometric evaluation of nutrition and PA assessment—online inter- and intrarater reliability.
Lafave et al [59]: Canada	Protocol for quasi-experimental study	ECEC centers	Total: 208 educators (intervention: 138; control: 50)	96.2%	40.05 (11.67)	•Food served in the center self-audit, eating environment/mealtime practices self-audit, nutrition education programming self-audit. •Mindful eating of educators. •Nutrition knowledge, attitude, and behaviors of educators. •Observation of nutrition environment in childcare. •Qualitative experiences of the nutrition environment. •PA environment self-audit. •Physical literacy knowledge, attitude, self-efficacy, and behaviors of educator and professional practices. •Objectively measured child PA levels. •Qualitative experiences of PA environment.
Lee et al [60]^e^: United States	RCT	Licensed childcare centers	Total: 30 ECE or services (intervention: 19; control: 11)	NR	Intervention group 47.7 (10.8); control group 49.6 (12.	• Knowledge and awareness of and adherence to California’s 2010 Healthy Beverages in Child Care Act.
Peden et al [61]: Australia	RCT	ECEC centers	Total: 104 educators	85%	NR	• Qualitative educator comments on the experience of the HOPPEL^m^ program.
Peden et al [62]: Australia	RCT	ECEC centers	Total: 112 educators	85%	NR	•Changes in center-level healthy eating practices assessed using the EPAO^n^ tool. •Changes in children’s PA assessed using ActiGraph GT1M and GT3X + accelerometers.
Reilly et al [63]: Australia	RCT	ECEC services	Total: 1024 ECEC services (intervention: 684; control: 342)	NR	NR	•Intentions to adopt the guidelines. •Awareness and reach of the guidelines. •Knowledge of the guidelines. •Implementation of the guidelines. •Barriers to implementing the guidelines.
Saunders et al [64]: United States	NR	Preschools	Total: 818 teachers	NR	NR	• Classroom implementation completeness (ie, provision of 300 minutes of PA opportunities) and fidelity (ie, achieving PA fidelity and social environment fidelity).
Ward et al [65]: United States	RCT	Full-time and part-time childcare center programs	Total: 33 ECE centers (intervention: 18; control: 15)	NR	NR	• Change in centers’ nutrition environments: (1) foods provided, (2) beverages provided, (3) feeding environment, (4) feeding practices, (5) menus, (6) education and professional development, and (7) nutrition policy.
Ward et al [25]^o^: Canada	RCT	Early childcare centers	Total: 191 (intervention: 102; control: 89)	NR	NR	•Healthy eating practices. •Perceived knowledge about fundamental movement skills. •PA practices.
Willis et al [66]: United States	Protocol for RCT	ECE centers	Total: 168 teachers	N/A	N/A	•Children’s dietary intakes and Healthy Eating Index scores. •Teachers’ dietary intakes and Healthy Eating Index scores. •Anthropometrics measurements (children and teachers). •ECE center nutrition environment. •PA (children and teachers using accelerometers). •ECE center PA environment.
Yoong et al [67]: Australia	Protocol for RCT	Childcare services	Total: 54 long-day-care services	N/A	N/A	•Mean number of food groups on childcare service menus that comply with dietary guidelines. •Proportion of services that comply with dietary guidelines for each of the 6 food groups. •Proportion of services that meet the recommended number of serves for all of the 6 Australian Guide to Healthy Eating food groups. •Child dietary intake. •Child BMI. •Child health-related quality of life.
Yoong et al [68]: Australia	RCT	Childcare centers	Total: 35 childcare centers	NR	NR	•Number of servings of the 5 core and discretionary food groups defined by the Australian Guide to Healthy Eating consumed in care. •Childcare educator–reported child diet quality. •Child BMI *z*-scores. •Child health-related quality of life. •Child diet outside of care.

^a^PA: physical activity.

^b^RCT: randomized control trial.

^c^NR: not reported.

^d^In Bruijns et al [45], participants included both preservice and in-service ECEs. Only data from in-service ECEs were reported, which aligns with our target population of educators in childcare centers.

^e^In Bruijns et al [45], Hazard et al [54], and Lee et al [60], participants included both center- and family-based childcare. For our purposes, we only included data from center-based childcare (eg, sample size). Gender and age are reported for all participants, as specific breakdowns were not provided.

^f^ELCC: early learning childcare center.

^g^ECE: early care and education.

^h^TRiPS: Teacher Tolerance of Risk in Play Scale.

^i^N/A: not applicable.

^j^WE PLAY: Wellness Enhancing Physical Activity for Young Children.

^k^MVPA: moderate-to-vigorous physical activity.

^l^ECEC: early childhood education and care.

^m^HOPPEL: Healthy Online Professional Program for Early Learners.

^n^EPAO: Environmental Policy Assessment and Observation.

^o^In Ward et al [25], the study included both in-person and online intervention groups. Our review focused only on extracting data from the online intervention group.

### eHealth Tool Characteristics

The 30 research articles included in the review identified 19 unique eHealth tools, with 12 categorized as intervention-based and 7 as both assessment and intervention tools. The characteristics of these eHealth tools identified in the white literature are summarized in Table 2, with additional details available in Multimedia Appendix 4. Among the 7 eHealth tools incorporating an assessment component, only 1 underwent evaluation of its psychometric properties. All eHealth tools were delivered via a web-based modality (7/7, 100%).

The characteristics of the eHealth tools identified in the gray literature are summarized in Table 3, with additional details provided in Multimedia Appendix 5. Of the 7 eHealth tools identified, all were intervention-only tools (7/7, 100%). Of these, 6 (86%) were delivered via a web-based modality, while 1 (14%) was delivered as a mobile phone app.

**Table 2:** Characteristics of eHealth tools from included studies identified in the white literature.

eHealth tool	Country: References	Type of eHealth tool	eHealth modality^a^	Target of eHealth tool	eHealth tool component	eHealth tool description	Length of the intervention	Theoretical underpinning
Create Healthy Futures Program	United States: [48]	Intervention	Web based	Nutrition	Healthy eating practices	Self-paced web-based intervention on promoting healthy eating behaviors for ECE^b^ providers	6 weeks	•Social Cognitive Theory •Social Ecological Model
CHEERS^c^	Canada: [58,59]	Assessment and intervention	Web based	Nutrition and physical activity	Nutrition and physical activity practices	Online educational modules and communities of practice to improve the nutrition and physical activity environment in ECECs^d^	10 months	•Social Cognitive Theory
EATS^e^	Australia: [27,42]	Assessment and intervention	Web based	Nutrition	Healthy eating practices	The web-based program supports center implementation of targeted healthy eating practices through self-assessment, feedback, and the development of an action plan	6 months	•Social Ecological Framework •Behavioral Change Wheel
FoodChecker	Australia: Kempler et al [56]	Assessment and intervention	Web based	Nutrition	Online menu planning	Web-based menu planning tool to support childcare services in planning healthy menus	NR^f^	NR
feedAustralia	Australia: [51,52,67,68]	Assessment and intervention	Web based	Nutrition	Online menu planning	Web-based menu planning program offering automated real-time assessment of childcare menu with feedback to support the planning of healthier menus	12 months	•Technology Acceptance Model •Theoretical Domains Framework
GO NAPSACC^g^	United States: [50,65]	Assessment and intervention	Web based	Nutrition and physical activity	Nutrition and physical activity practices	Suite of online tools to guide ECE programs through a 5-step process to improve their nutrition and physical activity-related practices, including (1) self-assessment, (2) goal setting and action planning, (3) implementation, (4) education and training, and (5) reassessment	4 months	•Social Cognitive Theory •DESIGN^h^ Procedure Framework •Theories of adult learning in public health practice •Behavior change techniques
GO NAPSACC Cares	United States: [66]	Assessment and intervention	Web based	Nutrition and physical activity	Nutrition and physical activity practices (center level) + educator’s personal diet and physical activity	The traditional GO NAPSACC program and embedded Staff Wellness website that focus on ECE personal healthy behavior change strategies including healthy eating, increased physical activity, and weight management	6 months	•Social Cognitive Theory •The Social Ecological Model
HOPPEL^i^	Australia: [61,62]	Intervention	Web based	Nutrition and physical activity	Nutrition and physical activity practices	Synchronous and asynchronous online professional development to promote physical activity and healthy eating	12 weeks	•Community of Practice •The Guskey model of teacher change
Healthy Start-Départ Santé	Canada: [25]	Intervention	Web based	Nutrition and physical activity	Nutrition and physical activity practices	Online modules to improve healthy eating and physical activity practices	4 hours	NR
InfaNET	United States: [49]	Intervention	Web based	Nutrition	Infant feeding practices	Bilingual (English and Spanish) website with childcare-specific infant feeding information	3 months	•Social Learning Theory
Munch & Move	Australia: [53]	Intervention	Web based	Nutrition and physical activity	Nutrition and physical activity practices	Professional development training for early childhood educators to support healthy eating and physical activity habits in young children	NR	•The Monitoring Framework
OutsidePlay-ECE risk-reframing intervention	Canada: [46,47]	Intervention	Web based	Physical activity	Outdoor/risky play	Fully automated web-based intervention to reframe ECEs’ perception of the importance of outdoor play and its inherent risks and promote a change in their practice in supporting children’s outdoor play in ELCC^j^ settings	Up to 100 minutes	•Intervention mapping process •Social Cognitive Theory
Online training on healthy beverage best practices	United States: [54]	Intervention	Web based	Nutrition	Healthy beverage practices	Self-paced online modules (English and Spanish) on healthy beverage best practices for childcare providers	English (29-minutes); Spanish (37-minutes)	NR
Online training on healthy beverage policy	United States: [60]	Intervention	Web based	Nutrition	Healthy beverage practices	Bilingual (English or Spanish) self-paced on-demand online training to increase knowledge and adherence of childcare providers to healthy beverage practices	30-minutes for the online training (+ with or without 6 months of online technical assistance)	•Implementation Science Framework •Humanistic Learning Theories
Online training to disseminate outdoor free-play information in relation to COVID-19 guidelines	Australia: [63]	Intervention	Web based	Physical activity	Outdoor/risky play	e-newsletter or animated video to increase ECEC service intentions to adopt an indoor-outdoor program for the full day and offer more time outdoors	e-newsletter (3 minutes); video (3.5 minutes)	•Model for Dissemination of Research •Interactive System Framework
SHAPES-D^k^	United States: [57,64]	Assessment and intervention	Web based	Physical activity	Physical activity practices	Self-assessment and online training modules to improve instructional physical activity practices and classroom social environment	12 weeks	NR
Tool to increase children’s vegetable intake and reduce food neophobia	Norway: [43,44]	Intervention	Web based	Nutrition	Online menu planning and feeding practices	Access to online menu recipes to include vegetables each week, with or without pedagogical tools (sensory lessons, meal practice, and feeding practices recommendations)	3 months	NR
TEACH^l^ e‐learning course	Canada: [45]	Intervention	Web based	Physical activity	Physical activity and sedentary behavior	e-Learning course in physical activity and sedentary behavior comprising 4 modules	2 weeks	•Social Cognitive Theory •The Theory of Planned Behavior
WE PLAY^m^	United States: [19,55]	Intervention	Web based	Physical activity	Physical activity practices	Online asynchronous modules to promote physical activity	4 weeks	Social Cognitive Theory The Theory of Planned Behavior Quality Implementation Framework
Online training on healthy beverage policy	United States: [60]	Intervention	Web based	Nutrition	Healthy beverage practices	Bilingual (English or Spanish) self-paced on-demand online training to increase knowledge and adherence of childcare providers to healthy beverage practices	30 minutes for the online training (+ with or without 6 months of online technical assistance)	Implementation Science Framework Humanistic Learning Theories

^a^eHealth modality (eg, web, app, or SMS text messages).

^b^ECE: Early Care and Education.

^c^CHEERS: Creating Healthy Eating and Active Environments Survey.

^d^ECEC: early childhood education and care.

^e^EATS: Child Care Electronic Assessment Tool and Support.

^f^NR: not reported.

^g^NAPSACC: Nutrition and Physical Activity Self-Assessment for Child Care.

^h^DESIGN: decide target behavior, explore determinants, select theory-based model, indicate objectives, generate education plans, and nail down the evaluation.

^i^HOPPEL: Healthy Online Professional Program for Early Learner.

^j^ELCC: early learning childcare center.

^k^SHAPES-Dissemination: Study of Health and Activity in Preschool Environments.

^l^TEACH: Training Pre‐Service Early Childhood Educators in Physical Activity.

^m^WE PLAY: Wellness Enhancing Physical Activity in Young Children.

**Table 3:** Characteristics of eHealth tools from included studies identified in the gray literature.

eHealth tool	Author/organization	Country: year	Participant information	Type of eHealth tool	eHealth modality^a^	Target of eHealth tool	eHealth tool description	Length of intervention
A Balanced Day: Tips and Guideline for Child Care Providers	Hastings Prince Edward Public Health	Canada: NR^b^	Childcare providers	Intervention	Web based	Physical activity	Online interactive modules (videos) on physical activity, sedentary behavior, sleep, and health messages for caregivers.	15 minutes
Boston Healthy Childcare Initiative	The Boston Public Health Commission	United States: NR	Early childhood educators	Intervention	Web based	Nutrition and physical Activity	Online training on evidence-based nutrition and physical activity best practices in early learning environments.	NR
Child Care Healthy Eating and Active Living Guidelines Training	Ottawa Public Health	Canada: 2015	Supervisors, childcare providers, and municipal cooks	Intervention	Web based	Nutrition and physical activity	Online training modules on healthy eating practices, environments, and physical literacy.	Part 1: Nutrition: 60 minutes; part 2: Active living: 30 minutes
Fostering Healthy Eating Habits	BC Provincial Health Services Authority—Interior Health	Canada: 2016	Childcare providers	Intervention	Web based	Nutrition	e-Learning course on healthy practices and environment.	1 hour
MyPlate on Alexa	United States Department of Agriculture	United States: 2020-2025	Everyone from parents and caregivers of babies starting at 4 months old through older adults (includes early learning professionals)	Intervention	App	Nutrition	An app that provides food and nutrition tips based on the Dietary Guidelines for Americans.	N/A^c^
Nourished and Active in Early Learning	University of Washington’s Center for Public Health Nutrition and Washington State Department of Health	United States: NR	Early learning professionals	Intervention	Web based	Nutrition	Online course on healthy eating and beverages comprising 6 modules, strategies to support healthy eating, and common challenges.	NR
Nourishing Beginnings	Dairy Farmers of Canada	Canada: NR	ELCC^d^ educators and directors as well as ELCC professors or program directors at colleges or universities.	Intervention	Web based	Nutrition	Online modules on healthy feeding practices	Module 1: 80 minutes; module 2: 70 minutes

^a^eHealth modality (eg, web, app, or SMS text messages).

^b^NR: not reported.

^c^N/A: not applicable.

^d^ELCC: early learning in childcare.

### Practices Targeted

**Figure 4:**
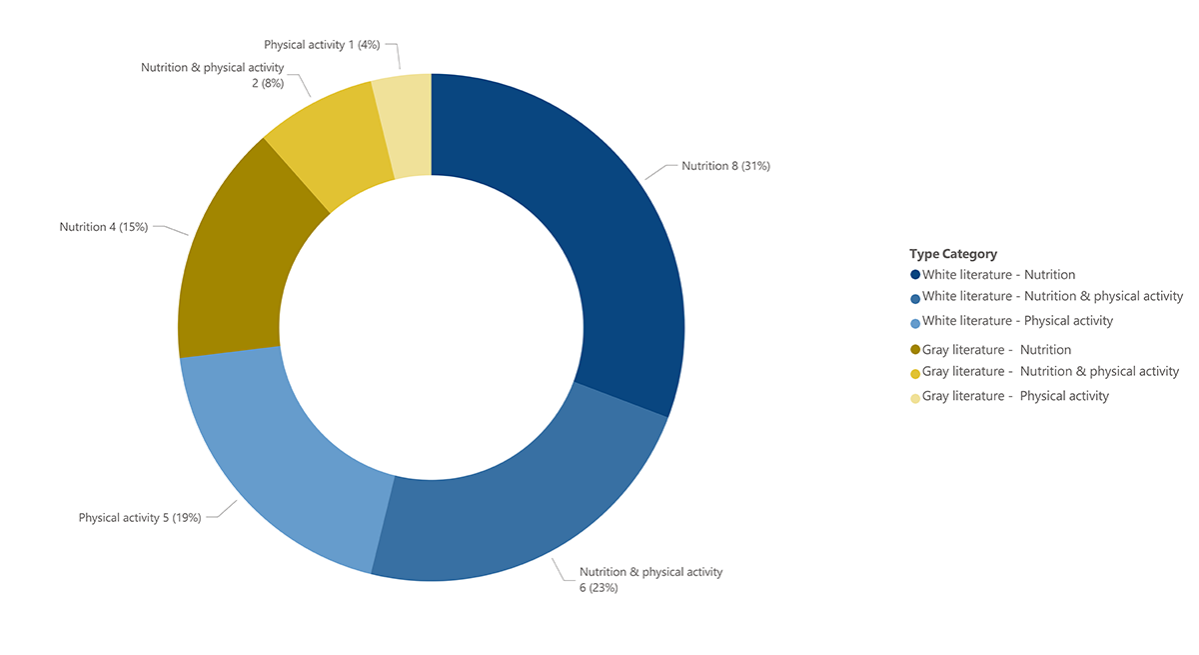
Target of eHealth tool for white and grey literature.

The majority of eHealth tools identified in the white literature (8/19, 42%) targeted nutrition practices, 6 of 19 (32%) targeted both nutrition and physical activity, and 5 of 19 (26%) exclusively addressed physical activity (Figure 4). The duration of intervention implementation ranged from 3 minutes to 12 months. The majority of eHealth tools identified in the gray literature targeted nutrition (4/7, 57%), followed by tools targeting both nutrition and physical activity (2/7, 29%), and 1 focused solely on physical activity (Figure 4). For both the white (Table 2) and gray literature (Table 3), eHealth intervention tools targeting nutrition primarily addressed best nutrition and feeding practices, healthy eating or beverage practices, and menu planning. eHealth tools targeting physical activity mainly focused on best physical activity practices, outdoor play, and the reduction of sedentary behaviors.

### Theoretical Framework of the eHealth Tools

**Figure 5:**
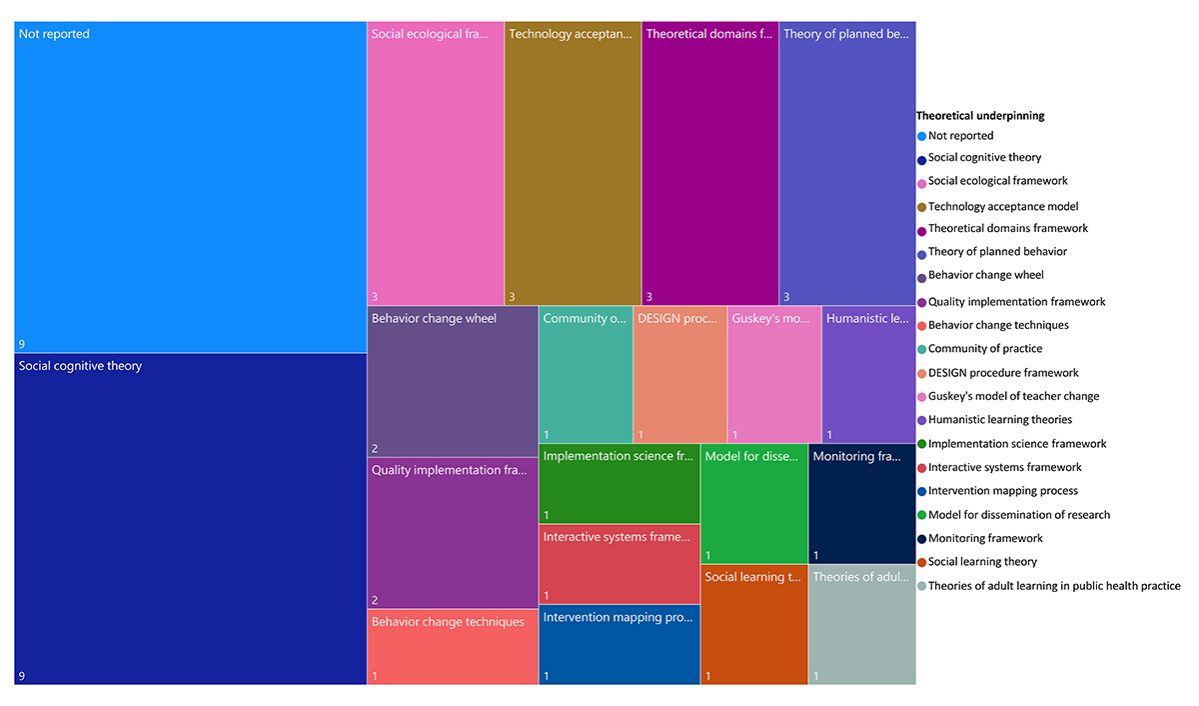
Theoretical underpinning from studies identified in the white literature search. DESIGN: decide target behavior, explore determinants, select theory-based model, indicate objectives, generate education plans, and nail down the evaluation.

Of the 30 research studies identified in the white literature, 21 reported the use of theoretical models (Multimedia Appendix 4). The most commonly cited theories include Social Cognitive Theory, Social Ecological Model, Theoretical Domain Framework, Theory of Planned Behavior, Behavior Change Wheel, and Quality Implementation Framework (Figure 5). None of the eHealth tools identified in the gray literature reported a theoretical underpinning (see Multimedia Appendix 5).

## Discussion

### Principal Findings

The purpose of this scoping review was to explore the available eHealth tools developed to assess or deliver interventions aimed at improving the nutrition or physical activity environments in ECEC settings. The results of this review provide insights into the digital tools available, outlining their methodological approach and characteristics.

Mapping the included white and gray literature identified 26 eHealth tools, highlighting a growing interest in leveraging digital tools to enhance nutrition and physical environments in early learning settings. Notably, all tools were web based except for 1 delivered via a smart device application. This finding is striking, given the widespread adoption of mobile health apps in health promotion interventions [69,70]. A likely explanation is that the nature and timing of the intervention may influence the choice of eHealth modality. Mobile apps are typically utilized in interventions requiring rapidly changing data and real-time feedback, such as step tracking or patient self-care and symptom management [71,72]. By contrast, interventions targeting nutrition and physical activity practices in educational settings often focus on gradual changes implemented over extended periods. Additionally, mobile apps are generally more expensive to develop and maintain than web-based tools and require regular updates [73]. Furthermore, most educational settings rely on web-based systems and have access to computers and the internet, making web-based platforms a more convenient option for educators while facilitating the implementation of interventions. Lastly, mobile-based interventions are often more prevalent among youth populations (children and adolescents) due to their capacity to incorporate gamification—a popular and engaging approach for health promotion interventions within this demographic [74].

The identified eHealth tools employed diverse approaches and strategies to enhance nutrition and physical activity practices. Most tools focused on professional development, offering online modules and self-paced training with both synchronous and asynchronous components to improve educators’ knowledge and behaviors related to best practices in nutrition and physical activity. Most of these tools (21/26, 81%) included educational videos to create engaging and interactive content, while just over half (15/26, 58%) incorporated quizzes or evaluation questions to reinforce learning and assess understanding. Additionally, a smaller proportion (10/26, 38%) provided technical support from experts or facilitators to offer guidance and enhance educator engagement. Research suggests that such human support can improve compliance and foster the adoption of new behaviors [75,76]. However, relying on human support may not be a sustainable approach due to the significant resources required and the challenges it poses for scalability [77]. A smaller proportion of tools (3/26, 12%) emphasized menu planning and provided tailored support to aid in the creation of healthier menus. Tools targeting physical activity primarily addressed best practices, highlighted the importance of outdoor and risky play, and aimed to reduce sedentary behaviors. Nutrition-focused tools concentrated on promoting best practices for nutrition and feeding while optimizing eating environments.

Less than one-third (8/26, 31%) of the identified eHealth tools addressed both nutrition and physical activity together. Evidence suggests that multibehavior interventions, which target multiple health behaviors simultaneously, are often more effective in driving meaningful change compared with interventions focused on a single behavior [78]. Given the interconnected nature of nutrition and physical activity, these behaviors play a crucial role in health and well-being [79,80] and are recognized as leading modifiable factors in the prevention of major chronic diseases [1,2]. Knowing that nutrition and physical activity tend to cluster together, addressing these behaviors collectively might enhance the effectiveness of interventions [11,81-84]. It has also been found that targeting both nutrition and physical activity behaviors can lead to synergistic effects on health outcomes [85-87]. Therefore, tools aimed at improving educators’ nutrition and physical activity practices or the nutrition and physical activity environments in early learning centers should target determinants of both behaviors simultaneously.

Another key finding is that, among the 7 eHealth tools incorporating an assessment component, only 1 reported psychometric properties testing. Psychometric evaluations, such as reliability and validity testing, are crucial as they indicate the quality of the tools, ensure their effectiveness for the intended purpose, and support their reproducibility and replicability [88]. The lack of reported psychometric testing may be due to some tools originally being developed in a pen-and-paper format before being adapted to an online format. In the original articles describing the pen-and-paper versions, psychometric testing was reported [89]. However, it is important to recognize that tool validity and reliability are not static characteristics; rather, they are assessments of the tool’s instrument scores within the context in which they have been evaluated [90]. It is therefore recommended to conduct and report updated psychometric testing when tools are adapted into digital formats to ensure that the validity and reliability of the results are maintained [91,92].

Among the 30 studies identified in the white literature, the majority (21/30, 70%) incorporated a theoretical framework in their design. The most frequently cited theory was the Social Cognitive Theory, which is commonly used in behavior change interventions, aligning with findings from other reviews [93-95]. A theoretical underpinning is a critical consideration when developing health promotion interventions. It serves as a blueprint for the study, structuring and guiding the intervention planning process, and also helps in understanding the factors that might influence behavior change and need to be targeted [96,97]. Previous reviews have indicated that health interventions grounded in theory are more effective [28] and are associated with positive significant outcomes, larger effect sizes [23,94,98], and the maintenance of behavior change [99]. Hence, future research designing eHealth tools should prioritize the use of theoretical underpinnings to increase effectiveness and ensure replicability. Regarding the gray literature findings, no reports on the theoretical underpinnings or reliability were identified for any of these tools. A possible reason for this could be that the gray literature often targets a general population audience, where the focus is on practical application, whereas in peer-reviewed literature, researchers look for evidence of theoretical grounding to evaluate and further refine or replicate these tools.

### Implications/Recommendations

Future research involving the development and implementation of eHealth tools in ECEC settings should emphasize the integration of theoretical frameworks, consider comprehensive multibehavior intervention approaches, incorporate community perspectives in the development process, and prioritize long-term sustainability and scalability, with a focus on implementation and efficacy assessment. Theoretical frameworks provide essential guidance in identifying key determinants of behavior and mechanisms for promoting change. Incorporating theory can enhance the effectiveness of eHealth tools, ensure consistent implementation, and facilitate replicability across different contexts. Multipronged eHealth tools designed to target both nutrition and physical activity can lead to more beneficial outcomes, as these domains are interconnected. To enhance implementation and adoption, it is essential to involve a diverse range of stakeholders, such as educators, parents, and health professionals, in the development process. This ensures that the unique needs and cultural context of the targeted eHealth tool users are addressed. Additionally, researchers should prioritize long-term sustainability and scalability when designing these tools, ensuring that interventions can be maintained and expanded across various settings over time. Finally, the resulting eHealth tools should undergo rigorous testing, including pilot trials, to assess their usability, feasibility, and effectiveness in achieving the intended outcomes. This integrated approach provides a holistic strategy for fostering healthier environments in ECEC settings.

### Strengths and Limitations

This review is the first to map and summarize the scope of available evidence on eHealth tools designed to assess or improve nutrition and physical activity environments in early learning settings. The findings from this review can guide future research on the use of eHealth technologies to promote healthy practices in childcare, ultimately contributing to improved children’s health behaviors and outcomes. Two librarians with expertise in scoping and systematic reviews assisted in developing and refining the search strategy. The methodology employed facilitated a systematic search of both white and gray literature, ensuring a comprehensive review of available evidence. However, there are some limitations to this scoping review that should be considered. We only considered studies published in English or French, which may have led to the exclusion of relevant research in other languages. Additionally, this scoping review focused solely on full-time day care centers, excluding family-based day care and after-school programs. The primary reason for excluding family-based day cares was the lack of formalized data, and after-school programs were not included as they typically do not cater to children aged 0-5 years. Another limitation encountered during the search for gray literature was the presence of paywalls and restricted access to certain tools, which hindered our ability to fully explore available resources.

### Conclusions

This scoping review explored the breadth of evidence on eHealth tools aimed at improving nutrition and physical activity environments in ECEC settings. Future research should conduct a systematic review to assess the effectiveness of these tools and identify the specific elements that contribute to a greater impact and sustained behavior change.

## Appendix

Multimedia Appendix 1 The Preferred Reporting Items for Systematic Reviews and Meta-Analyses Extension for Scoping Reviews checklist.

Multimedia Appendix 2 Search strategy for the databases (PubMed, Scopus, CINHAL Plus [EBSCOhost], ERIC [EBSCOhost], Embase [OVID]).

Multimedia Appendix 3 Data extraction forms for white and gray literature.

Multimedia Appendix 4 Extraction sheet results for white literature.

Multimedia Appendix 5 Extraction sheet results for gray literature.

## Abbreviations

ECEC: early childhood education and care
PRISMA-ScR: Preferred Reporting Items for Systematic Reviews and Meta-Analyses Extension for Scoping Reviews
