# Supplementary material for: Assessing and Enhancing Nutrition and Physical Activity Environments in Early Childhood Education and Care Centers: Scoping Review of eHealth Tools
Source: JMIR Pediatr Parent. 2025 Jan 22;8:e68372. doi: 10.2196/68372 (PMC11809617; doi:10.2196/68372)
Supplement: Multimedia Appendix 2 [file pediatrics_v8i1e68372_app2.pdf]

Multimedia Appendix 2. Search strategy for the databases (PubMed, Scopus, CINHAL Plus (EBSCOhost), ERIC (EBSCOhost), Embase (OVID)).

PubMed
Searched on October 4, 2023

Search	Query
#1	"early childhood education and care"[Title/Abstract] OR "day care*"[Title/Abstract] OR daycare*[Title/Abstract] OR "child care"[Title/Abstract] OR preschool[Title/Abstract] OR "licensed day care"[Title/Abstract] OR "long-term day care"[Title/Abstract] OR nursery[Title/Abstract] OR nurseries[Title/Abstract] OR kindergarten[Title/Abstract] OR "pre-school teacher*"[Title/Abstract] OR ECEC[Title/Abstract] OR educator*[Title/Abstract] OR "Child Care"[Mesh] OR "Child Day Care Centers"[Majr] OR "Child, Preschool"[Majr] OR "Nurseries, Infant"[Mesh]
#2	"e-health"[tw] OR ehealth[tw] OR m-health[tw] OR "online intervention"[tw] OR "online tool" [tw]OR "e-health tool"[tw] OR "digital intervention"[tw] OR "mobile health"[tw] OR "mobile application*"[tw] OR "mobile app"[tw] OR "mobile apps"[tw] OR internet-based[tw] OR "computer based"[tw] OR distance[tw] OR remote[tw] OR "web-based"[tw] OR "online systems"[tw] OR "mobile phone"[tw] OR "smartphone app"[tw] OR "Internet*"[Mesh] OR "Internet-Based Intervention*"[Majr] OR "self-assessment"[Mesh] OR "Mobile Applications*"[Mesh]
#3	"nutrition environment"[Title/Abstract] OR "Menu Planning"[Title/Abstract] OR "Nutrition Policy"[Title/Abstract] OR "Practice guidelines"[Title/Abstract] OR Physical activit*[Title/Abstract] OR "Physical play"[Title/Abstract] OR "active play"[Title/Abstract] OR "Beverages"[Mesh] OR "Nutrition Policy"[Mesh] OR "Diet*"[Mesh] OR "Exercise*"[Mesh] OR "feeding behavior"[Mesh]
#4	#1 AND #2 AND #3

Filter applied to search: limit to English and French language. 361 papers retrieved.

Scopus
Searched on October 4, 2023

Search	Query
#1	(TITLE-ABS-KEY) "early childhood education and care" OR "day care*" OR daycare* OR "child care" OR preschool OR "licensed day care" OR "long-term day care" OR "pre-school teacher*" OR ECEC OR educator* OR "Child Care" OR "Child Day Care Centers" OR "Child, Preschool" OR "Nurseries, Infant"

#2	"e-health" OR ehealth OR m-health OR "online intervention" OR "online tool" OR "e-health tool" OR "digital intervention" OR "mobile health" OR "mobile application*" OR "mobile app" OR "mobile apps" OR internet-based OR "computer based" OR distance OR remote OR "web-based" OR "online systems" OR "mobile phone" OR "smartphone app" OR "Internet*" OR "Internet-Based Intervention*" OR "Mobile Applications*"
#3	"nutrition environment" OR "Menu Planning" OR "Nutrition Policy" OR "Practice guidelines" OR Physical activit* OR “Physical play” OR “active play” OR "Beverages" OR "Nutrition Policy" OR "Diet*" OR "Exercise*" OR “feeding behavior”
#4	#1 AND #2 AND #3

Filter applied to search: limit to English and French language. 926 papers retrieved.

CINHAL Plus (EBSCOhost)
Searched on October 4, 2023

Search	Query
#1	Kindergarten OR Preschool* OR “early childhood education and care” OR “early childhood education” OR nurser* OR “day care” OR daycare OR educator OR childcare OR child care OR “early care and education” OR ((Child OR Day) N1 care) OR MH "Teachers" OR MH "Child Care Providers" OR MH "Child Day Care" OR MH "Day Care" OR MH "Child, Preschool"
#2	“ehealth” OR “e-health” OR “m-health” OR “mhealth” OR “online intervention” OR “online tool” OR “mobile application” OR “mobile app*” OR internet-based OR “computer based” OR remote OR “smartphone app” OR “mobile phone” OR “online training” OR “self-assessment” OR “online questionnaire” OR “online survey” OR “digital intervention” OR “web-based” OR “Online Education” OR (nutrition N2 online assessment) OR MH "Internet" OR MH "Internet-Based Intervention" OR MH "Mobile Applications" OR MH "Online Systems"
#3	“nutrition environment” OR “Menu Planning” OR Physical activit* OR “Physical play” OR “active play” OR “Healthy eating” OR MH "Nutrition Policy" OR “feeding behavior” OR “Health Policy” OR beverage* OR MH "Exercise" OR MH "Physical Activity" OR MH "Menu Planning" OR MH "Meal Preparation" OR MH “Health Promotion”
#4	#1 AND #2 AND #3 (Title OR Abstract)

Filter applied to search: limit to English and French language. 527 papers retrieved.

ERIC (EBSCOhost)
Searched on October 4, 2023

Search	Query
#1	Kindergarten OR Preschool* OR “early childhood education and care” OR nurser* OR “day care” OR daycare OR educator OR DE ”Early Childhood Teachers" OR DE "Preschool Teachers" OR DE "Child Care Centers" OR DE "Early Childhood Education" OR DE "Kindergarten" OR DE "Preschools" OR DE "Child Caregivers” OR ((Child OR Day) N1 care)
#2	“ehealth” OR “e-health” OR “m-health” OR “mhealth” OR “online intervention” OR “online tool” OR “mobile application” OR “mobile app*” OR internet-based OR “computer based” OR remote OR “smartphone app” OR “mobile phone” OR “digital intervention” OR “web-based” OR DE "Internet" OR DE "Online Surveys" OR DE "Online Courses" OR DE "Handheld Devices" OR DE "Web Based Instruction" OR DE "Nutrition Instruction" OR DE "Evaluation" OR DE "Intervention" OR (nutrition N2 online assessment)
#3	“nutrition environment” OR “Nutrition Policy” OR “Menu Planning” OR “feeding behavior” OR Physical activit* OR “Physical play” OR “active play” OR DE "Nutrition" OR DE "Lunch Programs" OR DE "Health Behavior" OR DE "Exercise" OR DE "Health Promotion" OR DE "Eating habit" OR DE "Teacher behavior" OR DE "Teacher attitude"
#4	#1 AND #2 AND #3 (Title And Abstract)

Filter applied to search: limit to English and French language. 394 papers retrieved.

Embase (OVID)
Searched on October 4, 2023

Search	Query
1	day care/ or *child day care/
2	child care/ or *infant care/ or *kindergarten/ or *nursery/
3	preschool child/
4	teacher/
5	(early childhood education and care).ti,ab.
6	(early care and education).ti,ab.
7	preschool.ti,ab.
8	preschool teacher.ti,ab.
9	educator.ti,ab.
10	ECEC.ti,ab.
11	(childcare or day care or daycare or (child adj3 care)).ti,ab.
12	telehealth/

13	web-based intervention/
14	online system/
15	mobile application/
16	ehealth.ti,ab.
17	e-health.ti,ab.
18	mhealth.ti,ab.
19	m-health.ti,ab.
20	mobile health.ti,ab.
21	online tool*.ti,ab.
22	(online adj2 (survey or assessment or questionnaire)).ti,ab.
23	nutritional assessment/
24	nutrition policy/
25	physical activity/
26	physical play.ti,ab.
27	active play.ti,ab.
28	beverage/
29	nutrition environment.ti,ab.
30	menu planning.ti,ab.
31	health promotion/
32	eating habit/
33	health behavior/
34	feeding behavior/
35	1 or 2 or 3 or 4 or 5 or 6 or 7 or 8 or 9 or 10 or 11
36	12 or 13 or 14 or 15 or 16 or 17 or 18 or 19 or 20 or 21 or 22 or 23
37	24 or 25 or 26 or 27 or 28 or 29 or 30 or 31 or 32 or 33 or 34
38	35 and 36 and 37

Limit applied: English and French language. 855 papers retrieved.
