# Supplementary material for: Assessing and Enhancing Nutrition and Physical Activity Environments in Early Childhood Education and Care Centers: Scoping Review of eHealth Tools
Source: JMIR Pediatr Parent. 2025 Jan 22;8:e68372. doi: 10.2196/68372 (PMC11809617; doi:10.2196/68372)
Supplement: Multimedia Appendix 3 [file pediatrics_v8i1e68372_app3.pdf]

Multimedia Appendix 3. Data extraction forms for white and grey literature.

Data extraction form (white literature)

Study ID (author, date)	
Study title	
Country	
Study aim	
Study design	
Study setting	
Participant information (age/sex)	
Inclusion/exclusion criteria	
Sample size	
Method of recruitment	
Type of eHealth tool (web-based, app-based, SMS)	
Tool components (what does it assess or support)	
Details of psychometric validation of tool	
Theoretical underpinning	
Outcome measures	

Data extraction form (grey literature)

Author/Organization	
Year	
Country	
Title of document	
Aim	
Design (if applicable)	
Setting (if applicable)	
Participant information (age/sex)	
Inclusion/exclusion criteria (if applicable)	
Sample size	
Method of recruitment (if applicable)	
eHealth modality (web, application, SMS etc.)	
Type of eHealth tool (assessment/intervention)	
Target of eHealth tool (Nut/PA or both)	
Tool components (what does it assess or support)	

Intervention duration	
Details of psychometric validation of tool (if applicable)	
Theoretical underpinning	
Outcome measures (Nutrition or Physical Activity)	
